# Molecular mechanisms defining penetrance of *LRRK2*-associated Parkinson’s disease

**DOI:** 10.1515/medgen-2022-2127

**Published:** 2022-08-12

**Authors:** Joanne Trinh, Emma L. Schymanski, Semra Smajic, Meike Kasten, Esther Sammler, Anne Grünewald

**Affiliations:** Luxembourg Centre for Systems Biomedicine, University of Luxembourg, Esch-sur-Alzette, Luxembourg; Institute of Neurogenetics, University of Lübeck, Lübeck, Germany; Department of Psychiatry and Psychotherapy, University of Lübeck, Lübeck, Germany; Medical Research Council (MRC) Protein Phosphorylation and Ubiquitylation Unit, School of Life Sciences, University of Dundee, Dundee, UK; Department of Neurology, School of Medicine, Dundee, Ninewells Hospital, Dundee, UK

**Keywords:** Parkinson’s disease, LRRK2, penetrance, genetic modifiers, Rab signalling, mitochondria, environment, toxin exposure

## Abstract

Mutations in *Leucine-rich repeat kinase 2* (*LRRK2*) are the most frequent cause of dominantly inherited Parkinson’s disease (PD). *LRRK2* mutations, among which p.G2019S is the most frequent, are inherited with reduced penetrance. Interestingly, the disease risk associated with *LRRK2* G2019S can vary dramatically depending on the ethnic background of the carrier. While this would suggest a genetic component in the definition of *LRRK2*-PD penetrance, only few variants have been shown to modify the age at onset of patients harbouring *LRRK2* mutations, and the exact cellular pathways controlling the transition from a healthy to a diseased state currently remain elusive. In light of this knowledge gap, recent studies also explored environmental and lifestyle factors as potential modifiers of *LRRK2*-PD. In this article, we (i) describe the clinical characteristics of *LRRK2* mutation carriers, (ii) review known genes linked to *LRRK2*-PD onset and (iii) summarize the cellular functions of *LRRK2* with particular emphasis on potential penetrance-related molecular mechanisms. This section covers *LRRK2*’s involvement in Rab GTPase and immune signalling as well as in the regulation of mitochondrial homeostasis and dynamics. Additionally, we explored the literature with regard to (iv) lifestyle and (v) environmental factors that may influence the penetrance of *LRRK2* mutations, with a view towards further exposomics studies. Finally, based on this comprehensive overview, we propose potential future *in vivo*, *in vitro* and *in silico* studies that could provide a better understanding of the processes triggering PD in individuals with *LRRK2* mutations.

## Clinical characteristics of *LRRK2*-associated Parkinson’s disease

Mutations in the *Leucine-rich repeat kinase 2* (*LRRK2*) gene are the most common monogenic cause of Parkinson’s disease (PD) [1]. They account for 1–2 % of PD cases and considerably more cases in certain populations such as North African Arab Berbers [2]. While idiopathic PD (IPD) is more common in men, this may not be the case for monogenic PD. In particular, *LRRK2*-PD seems to be more common in females. A recent meta-analysis of 64 studies and 32,452 patients reported a higher prevalence in women with a pooled relative risk of 1.22 (95 % confidence interval [CI] 1.14–1.30), and analyses of subgroups by mutation type suggested that this effect was restricted to G2019S mutations [3]. The cumulative incidence in Tunisian Arab Berber women with *LRRK2* G2019S is higher compared to that in males [4].

Systematic data on the clinical picture and course of *LRRK2*-PD are still scarce [3], rendering findings on clinical characteristics somewhat uncertain and to be interpreted with care.

*LRRK2*-PD resembles IPD in age at onset (AAO) and clinical signs, symptoms and progression [5], [6] with potential exceptions. Furthermore, there are different mutations known in *LRRK2*. A systematic review including comparisons of these mutations did not show clinical differences [7]. On the other hand, case numbers vary widely between mutations with p.G2019S being the most well known and common. Looking at clinical subtypes, i. e. tremor dominant, postural instability gait disorders and intermediate, the most frequent clinical subtype in *LRRK2*-PD is the postural instability gait disorder subtype [8]. Two studies found first a similar [8] and second a slower disease progression [9]. Interestingly there are several differences with regard to non-motor signs. *LRRK2*-PD patients have less impairment in cognition, smell and sleep [4], [10], [11], [12], [13], [14]. According to MDSGene, the most common cardinal feature was bradykinesia for p.G2019S mutation carriers (97 %, 152 out of 156), dyskinesia was reported in 66 % (69 cases of 105), dystonia in 39 % (24 of 61 cases) and motor fluctuations in 64 % (44 of 69 cases) of the included patients. However, these findings need to be interpreted with some caution as there are currently only two studies where longitudinal patient information was available [8], [9]. Cognitive decline occurred in 35 % (45 of 130 cases) and psychotic symptoms in 40 % (28 of 69 cases) of cases [7], [14]. In a meta-analysis focusing on cognitive and psychiatric features, dementia was relatively rare in *LRRK2*-PD compared to other monogenic forms of PD with the exception of Parkin [15] (58.8 % PINK1, 53.9 % SNCA, 50 % DJ1, 29.2 % VPS35, 15.7 % *LRRK2* and 7.4 % Parkin). However, cases with dementia have been reported [16]. Similar rates of depression between IPD and *LRRK2*-PD have been observed [17]. With a reported prevalence of 42 %, depression is, however, common in *LRRK2*-PD [15]. An examination of healthy *LRRK2* carriers and non-carriers detected increased UPDRS motor scores, more common urinary problems and fewer hours of sleep in carriers [18]. This may indicate either early signs or a *forme fruste* pointing towards incomplete penetrance and variable expression.

## Genetics and penetrance

Multiple pathogenic variants in *LRRK2* have been described: p.N1437H, p.R1441G/H, p.Y1699C, p.G2019S and p.I2020T [7]. These pathogenic variants are present at variable frequencies across the globe. Specifically, *LRRK2* p.R1441G is present at higher frequencies in familial PD in the Spanish Basque region (∼4–16.4 %), and the frequency of *LRRK2* p.R1441C is ∼4 % in Belgium [19], [20]. The most common *LRRK2* mutation is p.G2019S, with an estimated prevalence of ∼1 % in European familial PD populations and higher in North African Arab Berber or Ashkenazi Jewish populations (∼15–40 %). Importantly, the penetrance estimates are variable, initial estimates ranging from 28 % at age 59 years, 51 % at 69 years and 74 % at 79 years [6]. Since then, more estimates have been provided by different statistical means (Table 1). Of note, population-specific differences were seen between Tunisian Arab Berbers and Norwegian *LRRK2* p.G2019S carriers using a comparison of cumulative incidence [21]. Though not significantly different, a kin-cohort analysis showed that the risk of PD in non-Ashkenazi Jewish relatives who carry an *LRRK2* p.G2019S mutation was 42.5 % compared to 26 % in Ashkenazi Jews [22] (Table 1). The estimates vary but unequivocally show that there is reduced penetrance for *LRRK2* p.G2019S.

Genetic modifiers have been proposed to influence *LRRK2* penetrance or AAO. A variant located in the intronic region of *CORO1C* on chromosome 12 (rs77395454) was found to be associated with *LRRK2* penetrance and a suggestive association on chromosome 3 was found to be associated in an AAO model in a large cohort [23]. Previously, in a smaller homogeneous Tunisian Arab Berber population, *LRRK2* AAO was associated with a signal on chromosome 1, within *DNM3* [24]. Other linkage regions on chromosomes 1, 3, 4, 17 and 21 have been found to be linked to penetrance in G2019S families [24], [25]. *SNCA* and *MAPT* polymorphisms that were already significantly associated in PD GWAS have also been nominated as candidates [26], [27]. Besides the nuclear genome, mitochondrial DNA (mtDNA) and function has been shown to modify the affection status of *LRRK2* carriers [28]. The effect sizes from genetic studies thus far have been small. Still, only common SNP associations have been reported and thorough investigation of genetic modifiers using large-scale genome sequencing to elucidate the influence of structural variants, repeats, coding variants and regulatory regions is warranted.

## Cellular function of *LRRK2*

### The *LRRK2* protein and its functions

**Figure 1:**
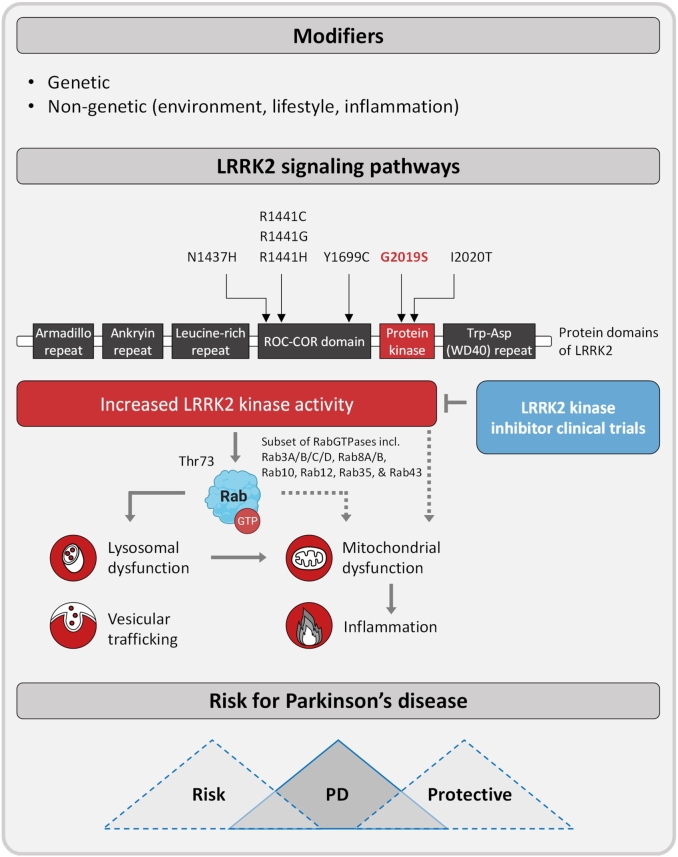
Scheme summarizing the levels of impact of genetic and non-genetic modifiers on the penetrance of mutations in Leucine-rich repeat kinase 2 (*LRRK2*). The most frequent mutation in *LRRK2*, G2019S, is situated in the kinase domain of the protein. Enhanced kinase activity due to G2019S increases the phosphorylation of a subgroup of Rab GTPases that were identified as substrates of *LRRK2*. Via Rabs, *LRRK2* acts on cellular functions such as lysosomal degradation and vesicular trafficking, which in turn may interfere with mitochondrial homeostasis and dynamics as well as immune signalling. How these molecular mechanisms control the transition of an *LRRK2* G2019S mutation carrier from healthy to diseased currently remains elusive. Nonetheless, significant efforts are being made to develop and advance *LRRK2* small molecule inhibitors into clinical trials as kinase inhibition is thought to slow the advancement of the movement disorder. Scheme adapted from Alessi and Sammler (2018) [43]. The figure was created using BioRender.com.

*LRRK2* is a large (2,527 amino acids), multifunctional protein with a catalytic core harbouring a Ras-like GTPase (Ras-of-complex [ROC] in tandem with a C-terminal ROC [COR] domain [ROC-COR GTPase domain]) and a second enzymatically active serine/threonine kinase domain. In addition, its N-terminal end comprises repetitive protein interaction motifs (armadillo, ankyrin and leucine-rich repeats [LRRs]) that are followed by its catalytic core and a tryptophan–aspartic acid repeat WD40 domain at the C-terminal end (Figure 1). There are only four proteins, including *LRRK2*, its shorter homologue *LRRK1*, death-associated protein kinase (DAPK) and a scaffolding protein (MASL1), that contain a ROC-COR GTPase domain [29]. The latter has been shown to be an active GTPase domain, albeit with low micromolar affinity so that *LRRK2* is found predominantly in the GTP-bound state [44]. GTP binding in turn is essential for *LRRK2* kinase function as point mutations introduced in the ROC domain such as Thr1348Asn that disrupt GTP binding abolish *LRRK2* kinase activity [45]. The kinase activity of *LRRK2* is of particular interest as all clearly disease-associated variants in *LRRK2* result in a gain of kinase function and kinase hyperactivation is a common mechanism in many diseases, including cancer, which has propelled the protein kinase family to become one the most important drug targets in the twenty-first century [46]. In fact, there are currently 62 FDA-approved small molecule kinase inhibitors targeting over 20 different kinases and much effort has gone into developing [47] and advancing *LRRK2* small molecule inhibitors in clinical trials (www.denalitherapeutics.com) (Figure 1).

**Table 1:** Estimates of *LRRK2* p.Gly2019Ser age-associated cumulative incidence and penetrance.

Ethnicity	Sample	Statistical analysis	Age range (cumulative incidence or penetrance)	Reference
Norway, United States, Ireland and Poland	13 *LRRK2* families 22 familial affected carriers	Proportion of affected/total carriers	50–70 (17–85 %)	[30]
French and North African families	2 *LRRK2* families 6 familial affected carriers	Not reported	55–76 (33–100 %)	[31]
Ashkenazi Jews	2,975 familial relatives of 459 probands	Kin cohort [32] *No relatives were genotyped for mutation: probability of carrying mutation was estimated	60–80 (12–24 %)	[33]
Ashkenazi Jews	22 affected carriers	Penetrance calculated from odds ratio	Lifetime risk = 35 %	[34]
Italian (UK Parkinson’s Disease Brain Bank)	36 familial affected carriers	Kaplan–Meier [35]	60–80 (15–32 %)	[36]*
Worldwide (mostly European)	133 *LRRK2* families 327 affected members	Kaplan–Meier [35]	59–79 (28–74 %)	[6]*
International multisite	22 familial affected carriers	Product limit survival estimate [35]	60–80 (30–55 %)	[37]*
Arab-Berber	72 affected carriers	Kaplan Meier [35]	60–80 (50–100 %)	[38]
European countries, mainly Italy	154 first-degree relatives and 190 second-degree relatives of 10 p.G2019S carrier probands	Kin cohort [32] *No relatives were genotyped for mutation: probability of carrying mutation was estimated	1st degree 60–80 (12–33 %)	[39]*
			2nd degree 60–80 (10–30 %)	
Northern Spain (Cantabria)	32 carriers	Kaplan–Meier [35]	60–80 (12–47 %)	[40]
Tunisian Arab Berber	266 *LRRK2* carriers from Tunisia	Kaplan–Meier and kin cohort [32], [35]	80 % by 70 years	[4]
Tunisian Arab Berber and Norwegian	220 affected *LRRK2* p.G2019S carriers, 6 unaffected from Tunisia.	Kaplan–Meier [35]	In Tunisia: 30 %, 61 % and 86 % of *LRRK2* p.G2019S carriers had developed parkinsonism by 50, 60 and 70 years of age.	[21]
	
	27 affected *LRRK2* p.G2019S carriers and 57 unaffected carriers from Norway		In Norway: 3 %, 20 % and 43 % had developed parkinsonism by 50, 60 and 70 years of age	
Tunisian, Norwegian, Israeli Ashkenazi Jewish	220 affected *LRRK2* p.G2019S carriers and 6 unaffected from Tunisia.	Kaplan–Meier [35]	In Tunisia: 30 %, 61 % and 86 % of *LRRK2* p.G2019S carriers had developed parkinsonism by 50, 60 and 70 years of age.	[41]
	
	27 affected *LRRK2* p.G2019S carriers and 57 unaffected carriers from Norway		In Israeli Ashkenazi Jews: 30 %, 61 % and 86 % of *LRRK2* p.G2019S carriers had developed parkinsonism by 50, 60 and 70 years of age.	
	
			In Norway: 3 %, 20 % and 43 % had developed parkinsonism by 50, 60 and 70 years of age	
Ashkenazi Jewish	2,270 relatives of 474 Ashkenazi Jewish Parkinson’s disease probands	Kin-cohort [32]	Risk of PD in relatives predicted to carry an *LRRK2* G2019S mutation was 0.26 (95 % CI 0.18–0.36) to age 80 years	[22]
Non-Ashkenazi Jewish	474 first-degree relatives of 69 non-Ashkenazi Jewish *LRRK2* p.G2019S carrier probands	Kin cohort [32]	Risk of PD in non-Ashkenazi Jewish relatives who carry an *LRRK2* p.G2019S mutation was 42.5 % (95 % CI 26.3–65.8 %) to age 80	[42]

Carriers refers to unrelated patients with *LRRK2* p.G2019S (unless otherwise specified). *Meta-analysis of previous reports. All studies are cross-sectional.

Pathogenic variants in the ROC GTPase (p.N1437H, the p.R1441 hotspot) and COR (p.Y1699C) domains suppress GTPase activity and promote GTP binding [48] and subsequently cause a 3–4-fold increase in *LRRK2* kinase activity [49], [50]. The common pathogenic p.G2019S and p.I2020T variants are located in the kinase domain and enhance *LRRK2* kinase activity moderately by about 2-fold by domain disruption [29].

Of interest is a cluster of constitutively phosphorylated serine residues (Ser910, Ser935, Ser955 and Ser973) located between the ANK and LRR domains that play a role in regulating 14-3-3 binding (Ser910/935) and cytosolic localization [51], [52]. These residues have received a lot of attention as potential biomarker sites as they are dephosphorylated in response to *LRRK2* inhibition [53], which has been widely used as *in vivo* pharmacokinetic markers, especially Ser935, for small molecule *LRRK2* kinase inhibitor compounds [54], [55], [56]. It is also clear that phosphorylation of these residues does not correlate with intrinsic *LRRK2* kinase activity [29], and emerging structural [57] and *LRRK2* kinase inhibitor profiling [58] studies suggest that these biomarker sites report on *LRRK2* conformation in an either inactive ‘open’ or active ‘closed’ conformation. Furthermore, various pathogenic variants such as p.R1441G, p.Y1699C and p.I2020T suppress the phosphorylation of Ser910 and Ser935 [57], [59] likely via mutation-induced conformational changes of *LRRK2*. In this context, microtubule association of *LRRK2* is also important as structural data have shown that pathogenic *LRRK2* variants that induce the closed active conformation become capable of microtubule binding in an ordered, oligomeric periodic manner, while the association of wild-type *LRRK2* or even the pathogenic p.G2019S variant with microtubules is significantly less efficient [57], [60]. Lastly, the serine residue 1292 of *LRRK2* deserves mentioning as it is a true *LRRK2* autophosphorylation site that correlates with intrinsic *LRRK2* kinase activity, but its low stoichiometry hinders the exploitation of Ser1292 as an *in vivo* biomarker site for *LRRK2* kinase activation [61].

### Rab GTPases and *LRRK2*

The reversible phosphorylation of proteins by kinases is a key regulatory mechanism that controls nearly every aspect of cellular life [62]. It was therefore a major step forward when a subgroup of Rab GTPases was unambiguously identified as endogenous substrates of *LRRK2* in 2016 – 12 years after the discovery that pathogenic variants in *LRRK2* cause PD [50]. In humans, there are over 60 Rab GTPases that are localized to distinct intracellular compartments and regulate membrane trafficking [63]. They exist in GDP-bound ‘inactive’ and GTP-bound ‘active’ states, with only the active state allowing the recruitment of cytosolic effector proteins via an α-helical Switch-II motif and subsequent formation of dynamic functional membrane domains for membrane trafficking, vesicle formation, movement along actin and tubular tracks and membrane fusion [29], [63], [64]. The *LRRK2* kinase phosphorylates Rab GTPases at a conserved Thr/Ser motif that lies at the centre of the Switch-II effector-binding domain [50]. As a result, the conformation-dependent interaction between Rab GTPases and their effector proteins including GDP dissociation inhibitors is perturbed with Rab GTPases becoming trapped at the membrane and generally inactive [63], [64].

**Figure 2:**
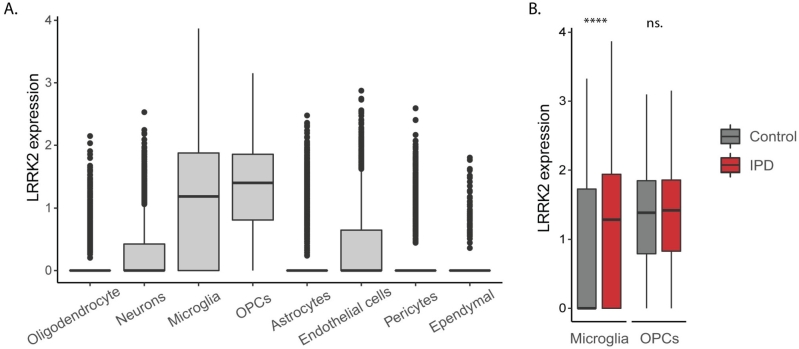
*LRRK2* gene expression in single cells from postmortem midbrain of idiopathic Parkinson’s disease (PD) patients and aged controls. We extracted *LRRK2* expression data from our previously generated single-nuclei RNA sequencing atlas of the human midbrain that is available on GEO under the accession number GSE157783 [65]. This analysis revealed highest levels of *LRRK2* in microglia and oligodendrocyte progenitor cells (OPCs). Comparison of the *LRRK2* mRNA abundance in these two cell types derived from IPD patients and controls revealed a disease-specific upregulation only in microglia.

*LRRK2* is predominantly localized in the cytosol and excluded from the nucleus, but about 10 % of *LRRK2* is membrane-bound, where it phosphorylates Rab GTPases [66]. Ten Rab GTPases have been shown to be endogenously phosphorylated by the *LRRK2* kinase including Rab3A/B/C/D, Rab8A/B, Rab10, Rab12, Rab35 and Rab43 [50] (Figure 1). Interestingly, some Rab GTPase effectors including Rab interacting lysosomal-like protein 1 (RILPL1) and RILPL2 preferentially bind Rab8 and Rab10 upon *LRRK2* phosphorylation, thereby interfering with ciliogenesis [50], [67], [68]. In the mouse brain, it has been shown that knock-in of the *LRRK2* kinase activating R1441G mutation impacts ciliary signalling in cholinergic interneurons in the dorsal striatum in terms of an attenuated neuroprotective response to a Sonic hedgehog signal received from dopaminergic neurons [67]. Another exciting discovery is PPM1H as the specific phosphatase that counteracts *LRRK2* signalling by dephosphorylating Rab GTPases [69]. Similar to pathogenic gain-of-kinase-function *LRRK2* mutations, knock-down of PPM1H suppresses primary ciliogenesis [69]. While future research will need to elucidate the role, effectors and downstream biology of the many other *LRRK2*-phosphorylated Rab GTPases as well as the link to PD, the development of tools and technologies such as phospho-specific monoclonal antibodies and sensitive proteomics mass spectrometry assays has aided in interrogating *LRRK2* kinase pathway activity *in vivo*, including in human peripheral blood [70], [71], [72], [73]. In fact, human peripheral blood neutrophils and monocytes lend themselves for interrogating *LRRK2* kinase pathway activity as they are relatively easy to obtain and both represent homogenous blood cell types with relatively high expression of both *LRRK2* and Rab GTPases. So far, peripheral blood neutrophils and monocytes have been used to demonstrate that PD patients carrying a pathogenic heterozygous VPS35 D620N mutation as well as carriers of the *LRRK2* R1441G variant have significantly increased *LRRK2*-dependent Rab10 phosphorylation levels [74], [75]. Such an enhancement has not been observed with the pathogenic *LRRK2* G2019S variant and this has likely to do with the sensitivity of the assay using Rab10 phosphorylation as a readout – as the *LRRK2* G2019S mutation activates *LRRK2* kinase activity only about 2-fold while the *LRRK2* R1441G and VPS35 D620N mutations result in a 3–4- and 4–6-fold increase, respectively [43]. In the future, it will be interesting to utilize alternative assays and readouts including other *LRRK2*-phosphorylated Rab GTPases for measuring *LRRK2* pathway activity.

### *LRRK2* in immune signalling

There is compelling evidence for a role of *LRRK2* in immune signalling (Figure 1). The protein is expressed in various immune cells including microglia [76], the resident macrophages of the brain [77]. When analysing an in-house single-nuclei RNA sequencing data set from postmortem IPD and control midbrain tissue, the highest levels of *LRRK2* transcripts were detected in oligodendrocyte precursor cells and microglia. However, only in the latter cell type, a disease-specific upregulation was observed (Figure 2). In line with these findings, *LRRK2* protein expression was detected in microglia derived from human induced pluripotent stem cells (iPSCs). The levels of *LRRK2* increased further after treatment with the immune stimulator interferon-gamma [78]. In this cellular system, *LRRK2* was shown to be involved in the recruitment of Rab8a and Rab10 to phagosomes, suggesting that the kinase functions at the intersection between phagosome maturation and recycling pathways [78]. Moreover, single-cell RNA sequencing of control iPSC-derived microglia revealed a strong induction of the mitochondrial antioxidant SOD2 upon treatment with α-synuclein pre-formed fibrils. This response was drastically reduced in microglia lacking *LRRK2*, strongly implicating the kinase in neuroinflammatory processes in the PD brain [77].

Yet, *LRRK2*’s immune action is not limited to the CNS. GWAS identified a variant in *LRRK2* that conferred an increased risk for Crohn’s disease [79] – an inflammatory bowel disease. Moreover, in Norwegian *LRRK2* families a high incidence of rheumatoid arthritis was reported [80]. Consistent with elevated *LRRK2* levels in B cells, T cells [81], macrophages [82], monocytes and neutrophils [71], the kinase is thought to regulate the immune response to pathogens [83]. *LRRK2* KO mice are more susceptible to intestinal *Listeria monocytogenes* infection than wild-type animals [84]. Similarly, *LRRK2*-deficient mice show increased bacterial colonization and reduced survival after infection with *Salmonella typhimurium* [85]. A mechanistic study in murine macrophages revealed that *LRRK2* kinase activity is crucial for the activation of the NLRC4 inflammasome during the host defence response [85].

Based on the current literature, scientists speculate that an overactivated *LRRK2* kinase may be beneficial during early life, protecting mutation carriers against infections [43], [83]. By contrast, with advancing age, chronic pro-inflammatory signalling may increase the permeability of the blood–brain barrier and facilitate microglial priming [43], [83], which in turn could aggravate neuronal degeneration. Thus, in accordance with the ‘second hit hypothesis’ in PD, the number and severity of infections that an *LRRK2* mutation carrier has to endure may define the penetrance of the movement disorder in this individual.

The ability of *LRRK2* to induce an immune reaction after infections may at least in part also explain the mitochondrial phenotypes observed in cellular models of *LRRK2*-PD. As an ancestor of a eubacterial endosymbiont [86], mitochondria harbour their own circular genome, which is characterized by low-level methylation [87]. In this way, mtDNA is distinct from nuclear DNA, and when released into the cytosol or extracellular space, it may be mistaken as foreign, triggering an autoimmune response against the patient’s own mitochondria. The research into the contribution of mitochondria to the pathogenesis in *LRRK2*-PD is manifold and will be briefly summarized in the next section.

### *LRRK2* and mitochondria

When investigating the subcellular localization of *LRRK2*, the protein was found to be associated with membranes, including the outer mitochondrial membrane [88]. A combination of co-immunoprecipitation, super-resolution microscopy and 3D virtual reality-assisted image analysis further uncovered that *LRRK2* interacts with subunits of the translocase of outer mitochondrial membrane (TOM) complex [89].

Inspired by its localization, researchers investigated mitochondrial morphology in cellular models of *LRRK2*-PD with diverging results. While early work showed elongated mitochondria in G2019S mutant fibroblasts [90], later phenotyping studies suggested a fragmentation of the mitochondrial network in patient cells [91], [92]. The latter observation is supported by functional experiments revealing a direct interaction between *LRRK2* and the mitochondrial fission protein DLP1 [88]. By modulating the abundance of DRP1 at the mitochondria, *LRRK2* kinase activity can regulate mitochondrial dynamics. Overexpression of wild-type *LRRK2* led to increased mitochondrial fission – a phenomenon that was further enhanced in the presence of PD mutations [93].

While the above-described network analyses in fixed patient cells only provided a static idea of the impairments caused by mutant *LRRK2*, mitochondrial motility assessments in *LRRK2* G2019S mutants were used to acquire further mechanistic insight. According to these analyses, *LRRK2* is involved in the removal of Miro1, which, together with Milton, anchors mitochondria to motors and microtubules for organellar transport [94]. In the presence of mutations in *LRRK2*, this link is stabilized by Miro1, thereby preventing the arrest and subsequent lysosomal degradation of dysfunctional organelles [94] (Figure 1).

Thus, there is evidence that *LRRK2* does not only interfere with mitochondrial clearance in this indirect fashion [88] (Figure 1). In fibroblasts from PD patients harbouring the G2019S mutation in *LRRK2*, increased mitochondrial–lysosomal co-localization was observed [91]. Moreover, analyses in patient fibroblasts harbouring the G2019S mutation and in HeLa cells overexpressing *LRRK2* G2019S revealed elevated protein levels of the autophagy markers p62 and LC3II [92], [95]. This result could either be suggestive of reduced lysosomal turnover or indicate accelerated mitophagy in *LRRK2* mutant cells. Interestingly, data to support both of these scenarios have been published. While Su and colleagues reported that overexpressed *LRRK2* G2019S phosphorylates Bcl-2 at Thr56, which exacerbates mitophagy in HeLa cells and primary rat neurons [95], Bonello et al. showed that Parkin-induced mitophagy is impaired in fibroblasts from patients carrying the G2019S mutation in *LRRK2* [96].

In line with an accumulation of damaged mitochondria, a multitude of studies described increased ROS generation and enhanced susceptibility to free radicals in cellular models of *LRRK2*-PD [97]. However, the oxidative stress phenotype may also be explained through *LRRK2*’s interaction with the mitochondrial antioxidant PRDX3. The peroxidase is phosphorylated by mutant *LRRK2*, which causes mitochondrial dysfunction and oxidative damage [97]. In addition, *LRRK2* G2019S was shown to increase the abundance of UCP2, which mediates mitochondrial uncoupling and proton leakage [92], [98]. This impact on respiratory chain function is further evidenced by reduced complex I activity [99] and oxygen consumption rates [100] in patient cells with the G2019S mutation.

Possibly as a result of increased oxidative stress, the mitochondrial impairments in *LRRK2*-PD extend to the mtDNA. Lesions in the mitochondrial genome were observed in neuronal cells derived from patients with the G2019S mutation [101]. Moreover, overexpression experiments in primary rat neurons showed that the mtDNA phenotype caused by *LRRK2* G2019S is midbrain-specific. Interestingly, treatment of these cells with an *LRRK2* kinase inhibitor sufficed to rescue the detected damage, suggesting that mtDNA disintegration in *LRRK2*-PD is kinase-dependent [102].

While the wide spectrum of mitochondrial alterations strongly implicate mitochondria in the pathology of the movement disorder, few functional studies focussed on the contribution of the organelles to the penetrance of *LRRK2*-PD. Our own research revealed that mtDNA major arc deletions are more abundant in manifesting compared to non-manifesting G2019S mutation carriers [28]. In addition, mtDNA replication was shown to be impaired in affected but not unaffected individuals harbouring *LRRK2* G2019S [103]. These disease-specific changes in mtDNA integrity and homeostasis appear to interfere with respiratory chain function as manifesting G2019S mutation carriers show lower complex I activity than their non-manifesting counterparts [99]. Increased mtDNA copy number and mitochondrial mass in patients with *LRRK2* G2019S may implicate impaired mitochondrial clearance as a penetrance-defining mechanism in these individuals [99]. Interestingly, in carriers of the G2019S mutation, also a PD-specific overexpression of *Nrf2* was detected. The transcription factor is part of the NF-E2-related factor 2–antioxidant responsive element (Nrf2–ARE) pathway, which is induced by ROS. Further strengthening this finding, Bakshi and colleagues performed a biomarker study in a large cohort of *LRRK2* mutation carriers and detected increased levels of the Nrf2 activator urate in affected compared to unaffected individuals [104]. Experiments in neurons from G2019S isogenic pairs highlight the contribution of the genetic background to the pathology of *LRRK2*-PD. In this study, in three iPSC lines, CRISPR/Cas9 gene editing was used to insert the G2019S mutation in *LRRK2* into a genetic control background. In another three lines, the G2019S mutation was corrected in a patient background. Various parameters of neuronal and mitochondrial morphology were then used in a cluster analysis, which resulted in a grouping by genotype. Moreover, an assessment of tyrosine hydroxylase abundance in the 3D cultures revealed that the generation of G2019S in the control background induced DA neuron demise. By contrast, *LRRK2* mutation correction in the patient background did not lead to a rescue of the DA neuron phenotype [105]. While these results could be explained by the genetic signature of a patient alone, they may also be reflective of the impact of environmental factors. Toxin exposure may exacerbate the molecular effects of increased *LRRK2* kinase activity in a patient in an irreversible manner (e. g. at the level of the epigenome and/or the mtDNA), thereby triggering the onset of PD in an individual.

## *LRRK2* and clinical lifestyle data

A combination of genetic and/or environmental factors influences PD susceptibility. For example, a meta-analysis has shown that smoking had a robust negative association with PD risk [106]. Furthermore, smoking is correlated with a later onset of motor and non-motor symptoms [107]. The causal protective relationship of smoking initiation has been supported by a recent Mendelian randomization study [108]. Although a direct causal relationship between PD onset and other lifestyle factors has yet to be established, coffee drinking is equally correlated with a reduced risk of PD [106]. Slower progression of motor and non-motor signs and symptoms has been shown for coffee consumers in a longitudinal study [109]. By contrast, with regard to environmental factors affecting PD, pesticide exposure is associated with an increased risk [106], [110]. The effects of environmental and lifestyle factors on AAO of *LRRK2* p.Gly2019Ser have yet to be thoroughly investigated. Reports have shown an association of smoking in *LRRK2* p.Gly2019Ser mutation carriers [111]: tobacco use is associated with later AAO in patients carrying the *LRRK2* p.G2019S mutation and the intensity and duration of smoking is correlated with AAO in these individuals. An effect of tobacco use was observed exclusively on non-motor symptoms but not on motor symptoms after adjustment for disease duration. Additional exploratory analyses on other lifestyle and environmental exposures revealed an interesting joint but independent effect for smoking and black tea drinking, indicating that a more complex interaction exists. Non-steroidal anti-inflammatory drugs (NSAIDs) have also recently been shown to correlate with *LRRK2* penetrance [112], [113]. The odds ratio for use of any NSAID was 0.34 (95 % CI 0.21–0.57); for ibuprofen it was 0.19 (0.07–0.50); and for aspirin it was 0.51 (0.28–0.91). A similar benefit was observed when NSAID treatment was five years before AAO [113].

Given the effects of lifestyle and environment on *LRRK2* [111] and the protective effects of smoking, caffeine and anti-inflammatory drugs on IPD [106], [109], there is ongoing interest in AAO, penetrance and other lifestyle factors in *LRRK2* parkinsonism (Figure 1).

## Environmental toxins as modifier of *LRRK2*-PD penetrance

Toxins are a subset of environmental factors that may influence PD susceptibility, yet the sheer number of potential toxins humans are exposed to in their daily environment renders this both an analytical and biomedical challenge. Estimates of chemicals in household use and/or on the market in significant amounts (i. e. tonnes of chemical) range between ∼70,000 to 350,000 [114], [115]; the largest open chemical databases now contain well over 100 million chemicals [116]. While many of the lifestyle factors mentioned above can be directly associated with particular chemicals (smoking and nicotine; coffee/tea and caffeine), nicotine and caffeine are only two of hundreds of other chemicals known to be present in cigarettes and tea/coffee, respectively (see references within [117]). Similarly, NSAIDs are also a large group of chemicals, with 159 NSAIDs mentioned in the Chemicals of Biological Interest (ChEBI) NSAID page (CHEBI:35475) alone (https://www.ebi.ac.uk/chebi) [118]. Pesticides are even more challenging, with over 3,000 agrochemicals (approximately synonymous with pesticides) listed in PubChem (3,101 dated 31 July, 2021) [116]. To date, epidemiology and exposomics studies have generally focussed on only a narrow range of dozens of known chemicals (toxins, often the so-called priority pollutants) or biomarkers in a targeted manner, which is unable to handle this sheer number of chemicals [119].

The rise of non-targeted high-resolution mass spectrometry (NT-HRMS) in environmental chemistry, metabolomics and thus also exposomics is opening the window to investigate further chemicals [119]. One of the biggest challenges in the exposome, which aims to not only measure the environmental exposure but also the biological perturbations that result, is the range of concentrations expected in samples. For example, biological signals in blood are at concentrations many orders of magnitude higher than the concentrations at which pesticides and other chemicals toxic in very low amounts could be expected to have an effect [120]. Some pharmaceuticals and other chemicals, such as caffeine and NSAIDs, can be closer to endogenous metabolites in concentration [120]. While information such as medical records and/or current blood samples could reveal present or past medication regimes of a patient, previous toxin exposure – potentially decades before disease onset in the case of PD – is much more difficult to capture. For instance, patients have been known to respond ‘no’ to questions regarding pesticide use, yet answer positively to ‘have you used Round-Up in your garden’ (the active ingredient, glyphosate, is a well-known pesticide) [117].

The definition of chemicals relevant for exposomics studies is an area of active research, since browsing the chemical space of tens of millions is not feasible. In one study, a set of 1,243 literature-mind neurotoxins (with more than five references) connected to distinct disease endpoints including PD was included in online databases and as an interactive Excel macro [117]. ‘CECScreen’ contains ∼70,000 structures and was an outcome of the Human Biomonitoring for EU (HBM4EU) project [121]; the Blood Exposome database contains ∼65,000 chemicals [122], while PubChemLite for Exposomics, a highly annotated subset of PubChem most relevant for exposomics studies, contains approximately 380,000 chemicals [123], including pharmaceuticals, pesticides, biomolecular pathways and all chemicals with associated disorders and diseases. Narrowing this down to a specific disease and gene reduces the window much further. The Comparative Toxicogenomics Database (CTD) [124] lists 78 chemicals interacting with *LRRK2* (http://ctdbase.org/detail.go?type=gene&acc=120892), with the most interactions belonging to several chemicals, including three pesticides often associated with PD: paraquat, maneb and rotenone (none of which, however, are approved for use in Europe), MPP+ (commonly associated with PD), as well as manganese and magnesium, benzo(a)pyrene and some biological molecules (lipopolysaccharides, adenosine triphosphate) and a steroid (corticosterone). These few chemicals alone would require several different analytical methods for detection [117]. Thus, the gap between literature associations [124], what can be detected in humans [120] and what is permitted and detected in environmental observations is still a challenge. However, the necessary tools to tackle these challenges are now available. A convergence of genetic-based disease interpretation and the influence of the environment via the concept of the exposome is likely to become a reality in the next few years as the fields slowly become aware of the mutual aims and challenges involved in merging the large amount of information available and forming concrete interpretations.

## Outlook

An unexpected outcome of recent major sequencing and gene identification efforts was the finding of a surprisingly large number of carriers of a putatively pathogenic mutation who remained free of the disease in question. This phenomenon of ‘reduced penetrance’ appears to have been substantially underestimated and the concept of protection against disease or delay of its AAO has been largely neglected within the genomic research community. The identification of such factors could have important implications for treatment and genetic counselling of patients. In the context of *LRRK2* parkinsonism, genetic and lifestyle penetrance modifiers can help in patient counselling and set the premise for future studies of endogenous protection.

The advent of iPSC technologies now permits the generation of patient-derived neuron and glia models, which allow studying the effects of drugs and pollutants at the endogenous level also in neurodegenerative disorders such as genetic PD. By contrast, investigating the effects of toxins in a systemic fashion will remain a challenge. While novel protocols for 2D or 3D co-culture systems and organoids are being developed, we are still far away from modelling the complexity of the brain in a dish. Still, large data sets that are being generated by applying especially ‘omics’ analysis methods to patient-derived cultures can inform *in silico* models of cellular function in response to stressors in PD. With regard to the human metabolome, a virtual database has already been generated ([125]; www.vmh.life) and efforts are being made to extend this work to specific cell types in PD [126]. However, currently, these models do not consider disruptions due to environmental insults, which may at least partially stem from the fact that the landscape of chemicals relevant in the pathogenesis of PD is not well defined.

Large data aggregators or knowledge bases such as PubChem can play a role in delineating the role of toxins in PD and even in the case of specific genes such as *LRRK2*. By integrating data from many resources, a single gene page provides an overview of several connections between the gene and chemicals, including from resources such as CTD [116], [127]. The data overview indicates quickly that literature observations conflict for the top chemicals mentioned in CTD such as benzo(a)pyrene and valproic acid. The top gene–chemical co-occurrences in literature listed on the *LRRK2* page in PubChem reveals several chemicals related to PD, some of those mentioned in CTD, and other chemicals of potential interest, which could be investigated pro-actively in patient samples via a suspect screening approach using NT-HRMS. Knowing the chemicals of interest in advance can assist in scoping and designing the appropriate analytical measurements. However, one disrupting factor in exposomics-based PD studies is the general disruption of the gut in PD patients compared with controls, which tends to lead to many perturbations related to gut dysfunction in blood/serum samples that may overwhelm the significance of any toxin signals [128], [129], [130] and thus interfere with the data interpretation. In this regard, other samples to capture patient exposure, such as dust samples [131] or surface and wastewater as a wider environmental proxy at the population level [132], are potentially of interest in the future to complement cohort studies and to advance research in patient-based cellular models as described above.
